# Biocomputational prediction of small non-coding RNAs in *Streptomyces*

**DOI:** 10.1186/1471-2164-9-217

**Published:** 2008-05-13

**Authors:** Josef Pánek, Jan Bobek, Karel Mikulík, Marek Basler, Jiří Vohradský

**Affiliations:** 1 Laboratory of Bioinformatics, Institute of Microbiology, Academy of Sciences of the Czech Republic, Prague, Czech Republic

## Abstract

**Background:**

The first systematic study of small non-coding RNAs (sRNA, ncRNA) in *Streptomyces *is presented. Except for a few exceptions, the *Streptomyces *sRNAs, as well as the sRNAs in other genera of the *Actinomyces *group, have remained unstudied. This study was based on sequence conservation in intergenic regions of *Streptomyces*, localization of transcription termination factors, and genomic arrangement of genes flanking the predicted sRNAs.

**Results:**

Thirty-two potential sRNAs in *Streptomyces *were predicted. Of these, expression of 20 was detected by microarrays and RT-PCR. The prediction was validated by a structure based computational approach. Two predicted sRNAs were found to be terminated by transcription termination factors different from the Rho-independent terminators. One predicted sRNA was identified computationally with high probability as a *Streptomyces *6S RNA. Out of the 32 predicted sRNAs, 24 were found to be structurally dissimilar from known sRNAs.

**Conclusion:**

*Streptomyces *is the largest genus of *Actinomyces*, whose sRNAs have not been studied. The *Actinomyces *is a group of bacterial species with unique genomes and phenotypes. Therefore, in *Actinomyces*, new unique bacterial sRNAs may be identified. The sequence and structural dissimilarity of the predicted *Streptomyces *sRNAs demonstrated by this study serve as the first evidence of the uniqueness of *Actinomyces *sRNAs.

## Background

Small untranslated RNAs (ncRNAs, sRNAs) with 50–1000 nts have been found to control a great variety of cellular processes in prokaryotic species [1]. Most bacterial sRNAs known to date act as post-transcriptional regulators by interacting with 5' leader regions of mRNAs, modulating mRNA stability and the ability of mRNAs to be translated [2]. The sRNAs are also known to interact with cellular proteins to modulate their activities. A well known example widely conserved in prokaryotes is the 6S RNA interaction which modulates σ^70^-holoenzyme activity [3]. Also, a few sRNAs (e.g. tmRNA) that serve housekeeping functions in streptomycetes have been identified [4].

The first bacterial sRNAs were discovered experimentally in *E. coli *[5-9]. Their common structural and functional features were elucidated and parameterized, and used for biocomputational prediction of novel sRNAs [10-13]. These features included conservation of intergenic regions (IGRs) in closely related bacterial species, the presence of predicted Rho-independent terminators and promoters, and genomic arrangement. Based on these features, the biocomputational searches identified new bacterial sRNAs in *E. coli *and closely related bacteria in the last few years.

The features originally used in biocomputational predictions in *E. coli *were applied to predict sRNAs in different bacterial species with various species-specific modifications. A consensus sequence for the σ^54 ^promoter was used as a criterion for sRNA prediction in *Vibrio cholerae *[14]. Similarly, a consensus sequence for the Fur repressor binding site was applied in *Pseudomonas aeruginosa *[15].

Also, other characteristic features were used for prediction of sRNAs. In cyanobacteria, the sRNAs were predicted based on computationally inferred conservation of RNA secondary structure [16]. In *V. cholerae*, the sRNAs were predicted based purely on IGR conservation and predicted Rho-independent transcriptional terminators, when specific criteria, such as a distances of either terminator or flanking genes from the conserved sequence were applied to the genomic arrangement of the predicted sRNA genes [17]. These criteria were generalized and used for sRNAs prediction in 10 diverse pathogens using a high throughput algorithmic approach [2]. The prediction also included bacterial species distantly related to *E. coli*; however, in total, only a few bacterial species distantly related to *E. coli *were studied. Also, the number of sRNAs known in *Streptomyces *is much smaller than in *E. coli*, where more than 100 sRNAs are known so far [1].

Very little is known about sRNAs in *Actinomyces*, particularly *Streptomyces*, the largest genus of *Actinomyces *with a high genomic GC-content. *Actinomyces *produce a variety of secondary metabolites, including antibiotics, and have a complex developmental cycle including growth phases from spores to vegetative forms. The intricate life cycle, together with an exceptionally large genome, suggests a complex regulatory machinery with a high number of sRNAs. However, so far, only three *Streptomyces *sRNAs (SRP bact, tmRNA and RNaseP bact a) and seven cis-regulating riboswitches were reported in the Rfam sRNA database [18]. Therefore, we aimed to predict more *Streptomyces *sRNAs, and in this study we present thirty-two *Streptomyces *sRNAs. The prediction employed IGR sequence conservation, co-localization of transcription termination factors and genomic arrangement. Expression of the predicted sRNAs was examined by microarrays and RT-PCR.

## Results

### sRNA prediction using Rho-independent transcription terminators

The presented prediction was based on the work of Argaman *et al*. [10], Wassarman *et al*. [19] and Rivas *et al*. [20]. It was based on sequence conservation in the intergenic regions (IGRs) of fully sequenced genomes, co-localized transcription terminators and genomic arrangement of the predicted sRNA genes.

In *Streptomyces*, two species have been fully sequenced to date, *S. coelicolor *and *S. avermitilis*, and their genomic sequences were used for the prediction. Using TIGR annotations, IGR sequences were identified in both genomes. There were 3753 and 4292 IGR sequences with lengths between 40 and 1000 nt identified in *S. coelicolor *and *S. avermitilis*, respectively. In these IGR sequences, the conservation was computed using BLAST [21]. BLAST databases were created for the *S. avermitilis *and *S. coelicolor *IGR sequences, and single IGR sequences were BLASTed against the database of the other species. The BLAST parameters were -r 1 -q -1 -G 1 -E 2 -W 9 -F "m D" -U -m 8.

For the conserved IGR sequences, co-localized Rho-independent terminators were identified. They had to start not farther than 50 nt downstream of the 3' end of the conserved sequences, oriented appropriately. The terminators were predicted by TransTermHP [22] with confidence > 75%. TransTermHP identifies the terminators by searching for a common mRNA motif: a hairpin structure followed by a short uracil-rich region. For each terminator, a score is assigned reflecting hairpin stability and related to the likelihood that it arose by chance.

For estimation of the BLAST E-value cut-off for significant sequence conservation, three different cut-off values, 1 × 10^-5^, 1 × 10^-10 ^and 1 × 10^-20^, were applied. They produced 1666, 1233 and 710 conserved IGR sequences, respectively. Among these, 63, 51 and 37 of the conserved IGR sequences had co-localized terminators. These decreased numbers showed that the screen was not sensitive to the terminator filter, but instead depended solely on the cut-off value, as more conserved sequences could have more co-localized terminators. Therefore, another indication for the cut-off estimate was required. To this end, the E-values between the three known *S. coelicolor *and *S. avermitilis *sRNAs, tmRNA, M1 RNA and 4.5S RNA, were computed. Their E-values were 2 × 10^-130^, 9 × 10^-94 ^and 2 × 10^-42^, respectively. In our dataset, we also identified two tRNAs that could also be considered as sRNAs with conserved structures. They had E-values of 6 × 10^-26 ^(tRNA ala) and 2 × 10^-27 ^(tRNA gly) (Table 1). These E-values were relatively much higher, while still representing strong conservation. If the cut-off was derived from the E-values of the three known sRNAs, sRNAs between 6 × 10^-26 ^and 2 × 10^-42 ^could be missed by the prediction. Therefore, the three known *S. coelicolor *sRNAs were BLASTed against the corresponding *E. coli *sRNAs. The E-values were 1 × 10^-14^, 6 × 10^-6 ^and 0.068 for M1 RNA, tmRNA and 4.5S RNA, respectively. Assuming that the sRNA conservation between *S. coelicolor *and *S. avermitilis *should be stronger than between *S. coelicolor *and distantly related *E. coli*, the BLAST E-value cut-off was chosen to be 1 × 10^-20^, lower than any of the three E-values.

**Table 1:** *Streptomyces *sRNAs predicted using Rho-independent terminators

ID#	Exp.†	Length	Strand*	Genomic coordinates	E-value	5' flanking gene distance	5' flanking gene termination&	RNAz probability @
4	++	78	← ⇒ ←	3082276..3082354	7 × 10^-25^	267		1
17	-0	161	← ⇒ ←	6702716..6702877	4 × 10^-53^	131		1
36	-0	72	← ⇒ →	7719646..7719718	2 × 10^-30^	274		0.98
73	++	53	→ ⇒ ←	6800040..6800093	2 × 10^-22^	122		0.91
84	-0	32	← ⇒ →	4153086..4153118	2 × 10^-27^	111		0.59
95	+-	312	← ⇒ →	6412268..6412579	3 × 10^-127^	10		0.7
96	-0	92	→ ⇐ →	6393104..6393196	3 × 10^-77^	0		-
114, 5S RNA	+0	132	← ⇐ ←	1916439..1916571	9 × 10^-47^	0		1
115	+-	246	→ ⇒ →	4530291..4530536	3 × 10^-118^	275	C-rich, 4530261..4530290	0.99
116	-0	122	→ ⇐ →	6266683..6266805	5 × 10^-77^	0		1
126	-0	118	→ ⇒ →	6144157..6144275	7 × 10^-60^	32	C-rich, 6143954..6144005	1
146	+-	80	→ ⇐ →	6005563..6005643	6 × 10^-31^	0		-
155	++	149	← ⇒ →	5922111..5922259	4 × 10^-57^	11		0.96
156	+-	93	→ ⇐ →	5912196..5912289	2 × 10^-32^	0		0.94
200	+-	150	→ ⇐ →	5647597..5647746	4 × 10^-127^	1		1
222	++	84	← ⇐ ←	5400596..5400680	4 × 10^-47^	25	?	0.9
234	-0	94	← ⇐ →	6033508..6033602	4 × 10^-67^	164		-
261, tRNA ala	+0	85	← ⇐ ←	3481828..3481913	6 × 10^-26^	0	C-rich, 3482039..3482068	0.99
270	++	118	← ⇒ ←	3506180..3506297	1 × 10^-16^	1		0.58
274	+-	189	← ⇒ ←	5040566..5040754	1 × 10^-50^	60		1
329, 4.5S	++	155	← ⇒ →	4456953..4457107	2 × 10^-42^	0		0.84
341	++	96	→ ⇐ ←	4375750..4375846	4 × 10^-40^	0	C-rich, 4375909..4375880	1
390	++	203	← ⇐ →	3933499..3933702	8 × 10^-74^	0		1
389	++	184	→ ⇐ →	3934660..3934844	3 × 10^-65^	0		1
413.1	++	338	← ⇒ →	3690627..3690965	3 × 10^-120^	412		1
413.2	-0	302	← ⇒ →	3690627..3691287	3 × 10^-120^	90		1
445	-0	192	→ ⇒ →	5076164..5076355	7 × 10^-67^	29	Rho-ind., 5076138..5076163	0.97
458	-0	210	← ⇐ →	5179518..5179728	6 × 10^-107^	0		0.67
462	-0	367	← ⇐ →	3321271..3321638	7 × 10^-156^	63		0.99
470, tmRNA	++	512	→ ⇐ ←	3226537..3227049	2 × 10^-130^	1	C-rich, 3227036..3227062	1
472	++	219	→ ⇐ ←	3208599..3208817	6 × 10^-51^	69	Rho-ind., 3208818..3208856	1
482, tRNA lys	+0	72	→ ⇐ ←	3079118..3079190	3 × 10^-50^	0	Rho-ind., 3079251..3079289	1
493	-0	48	← ⇒ ←	2984116..2984164	4 × 10^-65^	46		0.97
528	+-	170	← ⇒ ←	2646934..2647104	3 × 10^-27^	1		0.62
624	+-	159	← ⇒ ←	1765024..1765183	3 × 10^-21^	2		0.95
640, tRNA gly	+0	83	← ⇐ →	4469872..4469955	2 × 10^-27^	0		1
676	+-	308	← ⇒ →	1457688..1457996	1 × 10^-98^	23		1

† The first symbol stands for detection of expression by microarrays (expressed: '+', not expressed: '-'), the second one for RT-PCR confirmation of the expression (expressed: '+', not expressed: '-'). '0' stands for not applied.

* The double arrows represent sRNA gene, the arrows flanking genes. Right sided arrows show the complementary strand.

& Applicable only if the predicted sRNA and the gene flanking its 5' end are on the same strand. 'C-rich' and 'Rho-ind.' terms indicate the type of the transcription termination of the genes flanking 5' end of the predicted sRNAs. Genomic coordinates of the termination factors follow. The question mark for ID# 222 indicates a questionable C-rich stretch.

@ RNAz RNA-class probability. The higher this value, the more confident is the prediction of the functional RNA.

Using the E-value cut-off of 1 × 10^-20^, we obtained 710 conserved sequences in IGRs of the *S. coelicolor *and *S. avermitilis *genomes. Out of the 710 conserved IGR sequences, 37, with co-localized terminators, were considered to be potential sRNA genes (Table 1 and Table S1 in Additional file 1). Two sRNAs were predicted within one IGR (denoted as ID # 413.1 and 413.2 in Table 1), since two Rho-independent terminators were localized within a single IGR sequence. The 37 predicted sRNAs included two known *Streptomyces *sRNAs (tmRNA, 4.5S RNA), three tRNAs (tRNAs for alanine, lysine and glycine) and a 5S rRNA (Table 1) that were not included in the annotations used for the prediction. They therefore could not be excluded from the prediction and served as standards.

### sRNA prediction using alternative transcription termination

**Figure 1:**
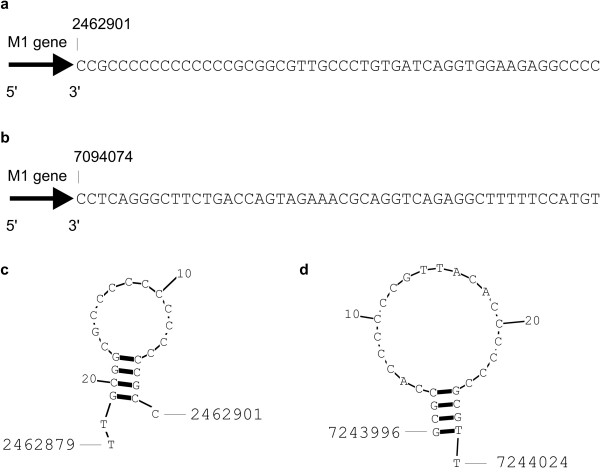
**Genomic organization of the *Streptomyces *M1 sRNA gene.** The *S. coelicolor *(a) and *S. avermitilis *(b) M1 genes are represented by arrows. The 50 nt sequences downstream 3' ends of the genes follow the arrows. The numbers show genomic coordinates. In (c), a structure of the C-rich stretch terminating the *S. coelicolor *M1 RNA is shown. In (d), another example of the C-rich stretch structure, terminating the ID # 60 predicted *S. coelicolor *sRNA, is shown. In (c) and (d), the numbers show genomic coordinates of 3' and 5' ends of the C-rich stretch.

Two known *Streptomyces *sRNAs (tmRNA, 4.5S RNA) were identified in the previous section using Rho-independent terminators. However, the third known *Streptomyces *sRNA, M1 RNA, was not found. This was due to its lack of a Rho-independent terminator. Instead of the terminator, we found a C-rich stretch with C-content of 73% (Figure 1a). It also had a structure with properties similar to the stem-loop structure of Rho-independent terminators [10] (Figure 1c). The termination of the M1 gene differed in *S. coelicolor *and *S. avermitilis*, as in *S. avermitilis*, a Rho-independent terminator was found at genomic coordinates 7094081 – 7094112 (Figure 1b). The terminator was predicted by TransTermHP with a high confidence (87%).

These data suggested that *Streptomyces *sRNAs might be terminated by a C-rich stretch stem-loop different from the classical Rho-independent terminator. We thus searched the conserved IGR sequences that lacked the co-localized Rho-independent terminators for the C-rich stem-loop. To this end, C-rich stretches were sought in 50 nt segments flanking the ends of the conserved IGR sequences. The stretches with G/C content > 75% and C content > 60% were folded using the RNAstructure program [23]. The structures were required to have at least 10 nt in the loop and 3 – 5 base pairs in the stem for them to be considered possible terminating factors. These properties were derived from the C-rich stem-loop of the M1 gene. Besides the M1 RNA gene, one gene was identified with such a C-rich stem-loop (ID # 60; Figure 1d). Together with the 31 sRNAs predicted using the Rho-independent terminator, the total of new predicted sRNAs was now 32.

### Genomic arrangement of the predicted sRNAs

Out of the 32 new predicted sRNAs and known *Streptomyces *sRNAs left in the dataset as standards (three known *Streptomyces *sRNAs, three tRNAs and a 5S RNA), 10 were localized on the same strand as the genes that flanked their 5' ends (5'-flanking genes) (Table 1). These 10 sRNAs needed to be distinguished from the 3' UTRs of mRNAs. Therefore, their 5'-flanking genes were inspected for transcription termination. Either Rho-independent terminators or C-rich stretches indicating the Rho-dependent termination were considered. The Rho-independent terminators were predicted by TransTermHP [22] and they were required to be localized in between the 5' end of the predicted sRNA and 100 nt upstream of the 3' end of the 5'-flanking gene, oriented appropriately. Three predicted sRNAs were found with such a genomic arrangement: two newly predicted sRNAs (ID # 445 and 472) and the tRNA for lysine (ID # 482) (Table 1).

To detect the Rho-dependent transcription termination, C-rich 30-mers were sought in between the 5' ends of the predicted sRNAs and 100 nt upstream 3' ends of the 5'-flanking genes. The C-rich 30-mers with C content between 35% and 60% have been reported to be required for one type of Rho-dependent transcription termination [24,25]. Here, 30-mers with C content > 60% were required, as *Streptomyces *have G/C rich genomes. Six sRNAs were identified with such a genomic arrangement: three predicted sRNAs (ID# 115, 126 and 341; Table 1) and three known *Streptomyces *sRNAs (tRNA ala, tmRNA and M1 RNA, Table 1 and 2).

**Table 2:** *Streptomyces *sRNAs predicted using an alternative transcription termination.

ID#	Exp.†	Length	Strand*	Genomic coordinates	E-value	5'-flanking gene distance	5' flanking gene termination#	RNAz probability @
60	++	252	← ⇒ →	7243744.. 7243995	2 × 10^-74^	17		0.99
544, M1 RNA	++	320	← ⇐ ←	2462901..2463220	9 × 10^-94^	2	C-rich, 2463157.. 2463177	1

† The first symbol stands for detection of expression by microarrays (expressed: '+', not expressed: '-'), the second one for RT-PCR confirmation of the expression (expressed: '+', not expressed: '-'). '0' stands for not applied.

* The double arrows represent sRNA gene, the arrows flanking genes. Right sided arrows show the complementary strand.

# Applicable only if the predicted sRNA and the gene flanking its 5' end are on the same strand. 'C-rich' stands for C-rich stretch of possible Rho-dependent transcrition termination. 'Rho-ind.' stands for a predicted Rho-independent terminator. The number following show the genomic coordinates of the termination factors.

@ In alignments with P < 0.5 a functional RNA is predicted. The higher this value, the more confident is the prediction.

### Experimental detection of expression of predicted sRNAs

Expression of the predicted sRNAs was examined by microarray analysis and RT-PCR. Internal oligonucleotides for the 32 predicted *Streptomyces *sRNAs were designed and spotted on microarray slides (see Materials and Methods). Specificity of the microarray signal was tested in two ways: 1. The housekeeping RNAs (5S rRNA and tRNAs for Ala, Arg, Gly, Ser and Lys) and two known *S. coelicolor *sRNAs (tmRNA and M1 RNA) were included in the experiment as standards; 2. The oligonucleotides for tmRNA, M1 RNA, 5S RNA and two predicted sRNAs (ID # 390 and 389) were designed with an increasing number (0, 2, 4, 6, 12, 24) of internal mismatches. As expected, the mismatches decreased the microarray signal by decreasing specificity of hybridization. Out of the 32 predicted sRNAs, 20 were found to be expressed (Table 1 and 2).

**Figure 2:**
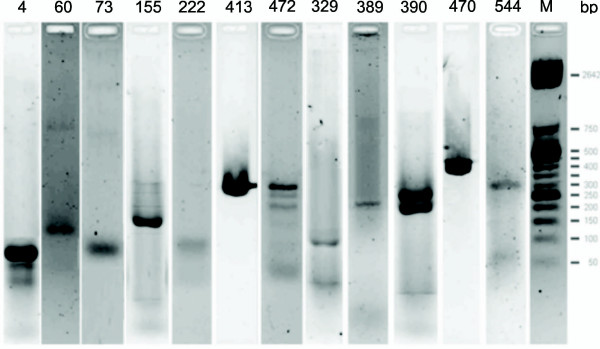
**RT-PCR of predicted *Streptomyces *sRNAs.** Numbers above the lanes show the ID # of the predicted sRNAs (as in Table 1 and 2). Standards are shown in the M lane at the right side of the figure.

The expression of sRNAs was further verified using RT-PCR. The 20 predicted sRNAs whose expression was detected by microarrays were reverse-transcribed using primers (18-mers) designed to match the predicted sRNA sequences immediately upstream of the transcription terminators. RNA samples were acquired in those growth phases that corresponded to the highest microarray signal. The RT-PCR recognized expression of 9 predicted sRNAs (Figure 2, Table 1 and 2).

### Structural and functional analysis of the predicted Streptomyces sRNAs

The predicted *Streptomyces *sRNAs were analyzed for conserved RNA structures using RNAz [26,27]. The analysis showed that 29 out of the total 32 predicted new *Streptomyces *sRNAs had a strongly conserved secondary structure (Table S4 in Additional file 1). For the three remaining (ID # 96, 146 and 234), no conserved structure was found or no prediction occurred because of too many gaps in their alignments. The 29 sRNAs were predicted to be functional sRNAs, for most of them (23) the probability was highly significant (> 0.9). The probabilities were included in the last column of Table 1.

To identify sequence similarities, the predicted sRNAs were computationally compared to sequences of known sRNAs of other species in Rfam database. The sequence similarity was computed using BLAST. The BLAST parameters were set as follows: -r 5 -q -4 -G 10 -E 6 -W 7 -F "m D" -U. The BLAST bit score cut-off for the Rfam database was 23.3 (the log_2 _of the size of the target database). The seven known *Streptomyces *sRNAs among the predicted sRNAs were identified correctly with bit scores > 100. Other BLAST hits (with bit scores between the cut-off and 100) were strongly ambiguous (except for one, as explained later), and they were most likely not biologically significant. Therefore, the biologically significant bit score cut-off was considered to be 100. No other BLAST hits with bit scores higher than this cut-off were found.

The predicted sRNAs were also matched against Rfam families using the Infernal package [28]. The Infernal method scores sequence and structure similarity at the same time. Moreover, Infernal uses consensus sequences and structures of the query sRNAs instead of single sequences. To this end, the sRNA sequences of *S. coelicolor *and *S. avermitilis *were aligned by ClustalW [29], and consensus structure was predicted using RNAalifold [30] from the alignments. Infernal was applied to the consensus structures in the local mode and without the HMM filter. The local mode was used since the *Streptomyces *sRNAs were supposed to have sequences and structures very different from most species in Rfam. Nevertheless, they might retain local similarities especially in the conserved functional sites. The local mode increases the probability of detection of such local similarities, as it allows for detection of partial similarities of the query model and target sequence. The HMM filter was not used in order to attain as sensitive a search as possible. The three known *Streptomyces *sRNAs, three tRNAs and the 5S RNA were among the predicted sRNAs that were identified correctly with bit scores > 50, and therefore the biologically significant bit score cut-off was estimated to be 50. However, no other hits with bit scores higher than this cut-off were found.

**Figure 3:**
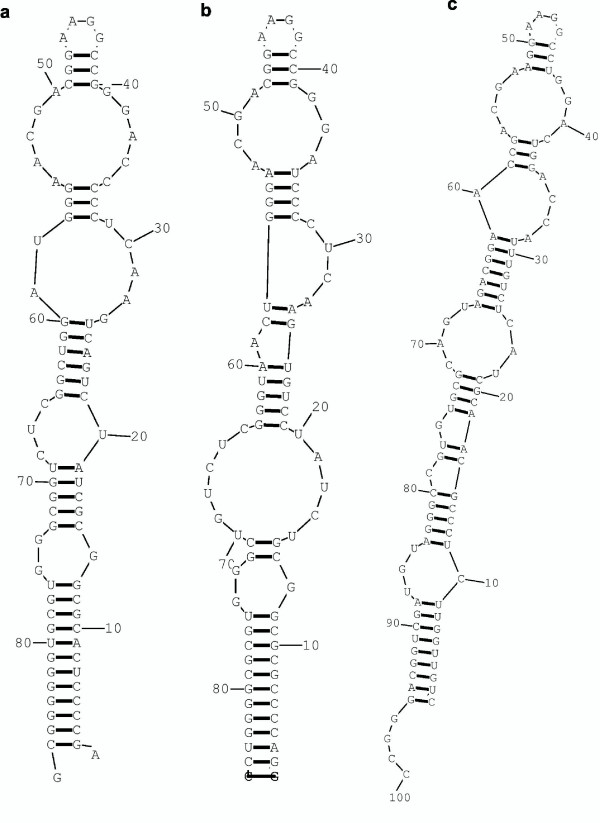
Structural similarity of the predicted *Streptomyces *sRNA ID # 329 (a) to 4.5S RNA of *Mycobacterium leprae *(b) and *E. coli *(c).

**Figure 4:**
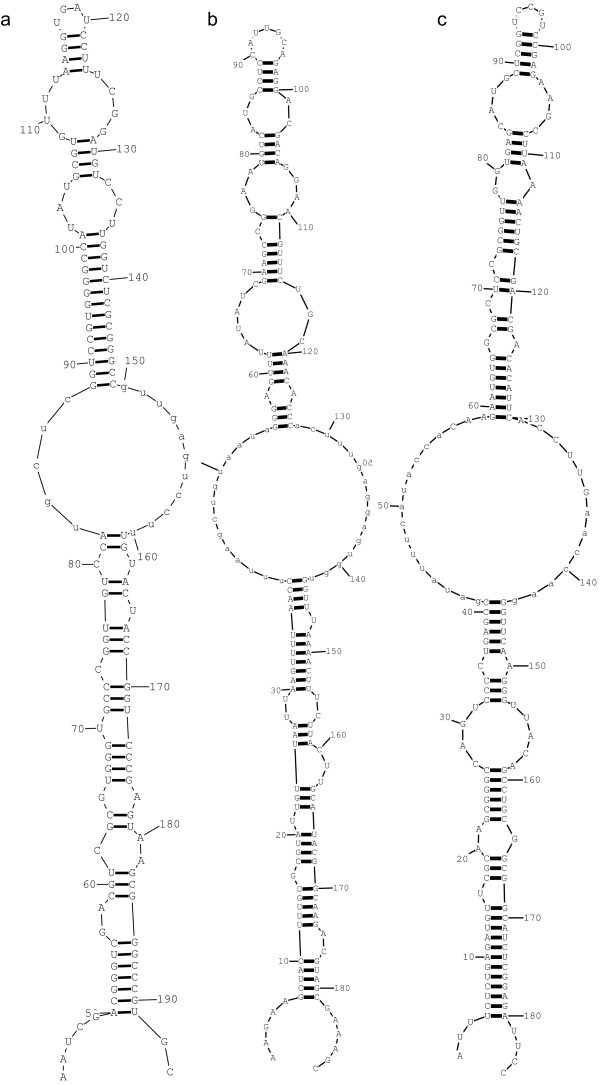
Structural similarity of the predicted *Streptomyces *sRNA ID # 389 (a) to 6S RNAs of *B. subtilis *(b) and *E. coli *(c).

We identified function features within the predicted sRNAs. The function features were derived from the known sRNAs of other species that were also expected to function in *Streptomyces*. They were M1, tm, 4.5S, spot42, 6S, oxyS, csrB, rprA, ryhB, dicF and micF RNAs [31]. The function features were identified based on expert knowledge, and were characterized by local sequences and local structures. They were mostly localized in the function sites of the sRNAs. The characteristic sequences and structures were identified in the sequences and structures of the predicted *Streptomyces *sRNAs. Significant hits were obtained in four cases: the three known *Streptomyces *sRNAs and the predicted sRNA ID # 389. The ID # 389 sRNA and the 4.5S RNA are shown in Figures 4 and 3, respectively, to demonstrate their analogous similarities to the known sRNAs of other species. In the case of *Streptomyces *4.5S RNA, the conserved function features were identified at nt 38 – 59 and nt 26 – 35 (Figure 3a). Analogously for the ID # 389 predicted sRNA, the conserved function features were identified at nt 75 – 90, 117 – 120, 124–128 and 148 – 161 (Figure 4a).

We therefore concentrated on the ID # 389 sRNA. It was the only unambiguous BLAST hit to the Rfam with a bit score (27.3) between the biologically significant and minimal significant bit score cut-offs. Its sequence corresponded to the only unambiguously identified promoter, when the promoters were sought manually for the predicted sRNAs. It was the *Streptomyces *σ^H ^transcription factor that corresponded to the σ^70 ^factor that is specific in *E. coli *for 6S RNA. Based on the promoter position, the length of the ID # 389 sRNA was determined to be 181 nt, which was similar to the lengths of known 6S RNAs (188 and 184 nt for *B. subtilis *and *E. coli*, respectively).

## Discussion

A systematic search for sRNAs in *Streptomyces *is reported. Thirty-two previously unknown *Streptomyces *sRNAs were predicted and their expression was experimentally examined by microarrays and RT-PCR. For most of the sRNAs, a high probability of functionality and high structure conservation were predicted using a computational analysis. Functional features were identified in one of the sRNAs, suggesting that it is a *Streptomyces *6S RNA.

In two of the predicted sRNA genes, a terminating C-rich stretch was found instead of a Rho-independent terminator. The C-rich stretch might be related to the Rho-dependent transcription termination mechanism [24,25]. However, the secondary structure of the C-rich stretch resembled the stem-loop structure of Rho-independent terminators. This similarity suggested that the C-rich stem-loop might be a *Streptomyces*-specific Rho-independent transcription terminator that differs from the "classical" Rho-independent terminator in details, but not in the overall structure. Supporting evidence includes two sRNA genes terminated by the C-rich stretch in *S. coelicolor *that are terminated by Rho-independent terminators in *S. avermitilis*. Considering the strong homology between *S. avermitilis *and *S. coelicolor*, one might also expect homologous transcription termination of these two genes. Whether the C-rich stem-loop structure is significant or not in transcription termination requires experimental verification. Nevertheless, the C-rich stem-loop structure was successfully used in the presented prediction.

In the previous studies [10,19,20], promoters served for verification of the 5' ends of the predicted sRNAs. We could not use the promoters because a large number of *Streptomyces *promoters are unknown [32,33]. Also, the binding site sequences of 155 known Streptomyces promoters [34] were found to be ambiguous. Therefore, instead of promoters, we checked for transcription termination of 5' flanking genes, i.e. genes that flanked 5' ends of the predicted sRNAs, when the predicted sRNAs and their 5' flanking genes were localized on the same strand. Not only was such a criterion useful for distinguishing the predicted sRNAs from the 3' UTRs, but it also helped to estimate the length of the predicted sRNAs, when the promoters were unavailable.

In-depth analysis was carried out to identify the sequence and structure similarity of the predicted *Streptomyces *sRNAs to known sRNAs. However, a significant similarity was identified for only one of the novel predicted sRNAs. Thus, 24 predicted sRNAs (out of the total 32) were found to be dissimilar from the known sRNAs. The number was surprisingly high, suggesting strong sequence and structural dissimilarity of *Streptomyces *sRNAs. The dissimilarity of the 24 predicted sRNAs served as the first evidence of the uniqueness of *Streptomyces *sRNAs. The uniqueness most likely is related to the phenotypic and genomic dissimilarity of *Streptomyces*.

In the presented prediction, the experimental detection of expression of the predicted sRNAs was accomplished by the combination of microarrays and RT-PCR. These methods were used instead of Northern blots, where the Northern blot had been employed in most of the previous predictions [1]. The combination of microarrays and RT-PCR proved to be more sensitive than the Northern blot and had a much higher throughput. This was useful, as a relatively high number (39) of predicted sRNAs in several *Streptomyces *growth phases were required to be examined. A similar approach has been used before to overcome the poor sensitivity and low throughput of conventional technologies including the Northern blot [35]. When Northern blot was applied here, expression of only 5S RNA and tmRNA was detected (not shown). 5S RNA and tmRNA are expressed during the entire life cycle of *Streptomyces*, and therefore available in amounts sufficient for detection by Northern blot [36]. However, the other predicted sRNAs might be expressed in short specific growth phases in amounts that were under the detection threshold of the Northern blot. One may argue that agreement between the lengths of the Northern blot transcripts and lengths of the predicted sRNAs validated the prediction and that the PCR transcripts have lengths less similar to the lengths of the predicted sRNAs. However, it was shown here that the length differences allow for a clear discrimination of the transcripts from the 3' UTRs of the mRNAs. This was demonstrated in Figure 2. The discrimination of sRNAs from 3' UTRs also was addressed computationally as the prediction required the transcription termination to be present in the genes flanking the 5' ends of the predicted sRNAs.

Results of the presented study suggested that the rules valid for sRNA prediction in other bacteria could be used only partly in *Streptomyces*. This is most likely a result of two factors: 1. the phenotypic and genomic uniqueness of *Streptomyces*, 2. the lack of data, such as binding sites of promoters and/or repressors. In light of the two facts, further (functional) characterization of the predicted sRNAs would be spurious. Namely, the function prediction, based on function of flanking genes, might be very risky in *Streptomyces*, as *Streptomyces *have a relatively complex genomic organization. Therefore, the functional characterization requires wet-lab experiments.

The presented prediction is – to our knowledge – the first systematic search for sRNAs in *Actinomyces*. Function of the predicted sRNAs will be characterized experimentally. It may be expected that unique RNA functions will be revealed, due to the genomic and phenotypic uniqueness of *Actinomyces*. To identify remaining sRNAs, *Streptomyces *genomes sequenced in the near future and other *Actinomyces *genomes will be used (genome sequence of *S. griseus *is about to be finished, *S. ambofaciens *is almost 75% finished). Also, new prediction criteria and experimental validation delivered by the presented study will be employed.

## Methods

### Computational prediction

*S. avermitilis *and *S. coelicolor *genomic sequences were imported from the NCBI ftp site [40]. ORF annotations were obtained from TIGR Comprehensive Microbial Resource [41], including tRNAs and rRNAs ORFs. The computation and algorithms used in this study was made using MATLAB and Bioinformatics toolbox [37].

### Experimental verification

#### Strains, growth conditions and RNA isolation

Cultures of *S. coelicolor *A3(2) strain M145 were grown in NMMP liquid medium [38] at 28°C with shaking at 150 rpm, either to exponential phase (24 hours), transition into stationary phase correlating with the beginning of antibiotic production (48 hours), or late stationary phase (6 days). Exponentially growing cells were inoculated on PPS solid agar medium (%, *W/V*: malt extract 1, yeast extract 0.4, glucose 0.4, agar 2; pH 7.2) and cultivated until sporulation (10 days). Samples (0.3 g wet weight) from all different stages of cell development were homogenized with glass beads (0.1 mm) in 1 ml RNA Blue (TopBio), and were processed four times for 40 seconds each in the FastPrep machine at setting 5.5 with cooling between the stages. Total RNA was isolated using RNA Blue (Top-Bio) according to the manufacturer's protocol. Each RNA sample was treated with 5 U of RQ1 RNase-free DNase I (Promega) at 37°C for 15 min, and RNA was precipitated with 2.5 vol of ethanol. Washed RNA with 75% ethanol, it was solubilized in 10 mM Tris, pH 8.0, and the RNA concentration was estimated by measuring absorbance at 260 nm.

#### Microarray analysis

Internal DNA oligonucleotides (Table S1 in Additional file 1) were designed for the 52 predicted sRNAs using the Primer3 program [39] with general conditions set. The oligonucleotides were spotted on microarray slides. Cy3/Cy5-labelled cDNA was synthesized from 15 μg of the RNA sample using random hexamers and Superscript II reverse transcriptase (Invitrogen). To detect expressed transcripts, the spotted oligonucleotides were hybridized with cDNA for 4 h at 58°C. Hybridized slides were washed in 1× SSC, 0.2% SDS (10 min); 0.1× SSC, 0.2% SDS (10 min); and 0.1× SSC (1 min) according to the Array-It protocol, and scanned on an Affymetrix GeneArray Scanner. Hybridization efficiency was established by means of GeneChip 3.1 software.

#### RT-PCR

From the transcripts, expression of which was detected by microarrays, 13 were chosen randomly for verification by reverse transcription and PCR amplification (*RT-PCR*). First strand cDNA was synthesized from 15 μg of total RNA harvested at different stages of cell growth (Superscript II, Gibco/BRL) using 18-mer primers designed to be complementary to the part preceding the terminal hairpin of the applicable sRNA. The resulting cDNA was polyadenylated on its 3' end by terminal transferase (400 U; Roche). PCR (25 μL), containing the corresponding primer (sense), T_(18)_VN primer (anti-sense) and the 3' polyA-cDNA as a template, was performed using Taq DNA polymerase (1,25 U; Fermentas).

PCR cycling consisted of a single incubation at 95°C for 5 min, followed by 38 cycles of 95°C for 30 s, 38°C for 30 s and 72°C for 1 min with a final single extension step of 72°C for 7 min.

## Authors' contributions

JP and KM initiated and conceived the study. JP designed and carried out the biocomputational prediction, functional analysis of the predicted sRNAs, and wrote the manuscript. KM participated in functional analysis of the predicted sRNAs based on expert knowledge. JB designed and carried out the experimental validation of expression of the predicted sRNAs. MB participated in the experimental validation. JV participated in coordination of the study and writing the manuscript. JV and KM provided critical feedback for the final version of the manuscript. All authors read and approved the final manuscript.

## Supplementary Material

Additional file 1 Supplementary tables Click here for file
