# Supplementary material for: Biocomputational prediction of small non-coding RNAs in Streptomyces
Source: BMC Genomics. 2008 May 13;9:217. doi: 10.1186/1471-2164-9-217 (PMC2422843; doi:10.1186/1471-2164-9-217)
Supplement: Additional file 1 — Supplementary tables [file 1471-2164-9-217-S1.doc]

Table SI. Further characteristics of the *Streptomyces* sRNAs predicted using Rho-independent terminators

| sRNA ID # | Strand* | Flanking genes annotations *** | Terminator coordinates** | Conserved sequence coordinates | IGR coordinates |
| --- | --- | --- | --- | --- | --- |
| 4 | ← ⇒ ← | secreted pectate lyase / decarboxylase | 3082354..3082381 | 3082276..3082391 | 3082009..3082430 |
| 17 | ← ⇒ ← | nitrite/sulphite reductase / acetyltransferase | 6702877..6702903 | 6702716..6702920 | 6702585..6702969 |
| 36 | ← ⇒ → | tRNA Ser / tRNA Arg | 7719718..7719757 | 7719646..7719707 | 7719372..7719817 |
| 73 | → ⇒ ← | hypothetical protein / luxR | 6800093..6800116 | 6800040..6800161 | 6799918..6800241 |
| 84 | ← ⇒ → | hypothetical protein / ATP-binding protein | 4153118..4153141 | 4153086..4153155 | 4152975..4153189 |
| 95 | ← ⇒ → | uknown / unknown ATP/GTP binding protein | 6412579..6412603 | 6412258..6412683 | 6412257..6412682 |
| 96 | → ⇐ → | putative membrane protein / probable transcriptional regulator | 6393082..6393104 | 6393136..6393196 | 6393074..6393195 |
| 114, 5S RNA | ← ⇐ ← |  | 1916399..1916439 | 1916434..1916571 | 1916356..1916570 |
| 115 | → ⇒ → | MarR transcriptional regulator / 16S rRNA | 4530537..4530570 | 4530215..4530581 | 4530126..4530669 |
| 116 | → ⇐ → | unknown / dapA, dihydrodipicolinate synthase | 6266638..6266683 | 6266556..6266805 | 6266533..6266804 |
| 126 | → ⇒ → | unknown / solute binding lipoprotein | 6144275..6144318 | 6144157..6144348 | 6144125..6144349 |
| 146 | → ⇐ → | ilvN, acetolactate synthase small subunit / ilvC, acetohydroxy acid isomeroreductase | 6005535..6005563 | 6005531..6005643 | 6005504..6005643 |
| 155 | ← ⇒ → | pep1A, possible alpha-amylase / glgP, possible glycogen phosphorylase | 5922264..5922282 | 5922100..5922259 | 5922099..5922320 |
| 156 | → ⇐ → | trxA2 / helicase | 5912171..5912196 | 5912156..5912289 | 5912134..5912288 |
| 200 | → ⇐ → | uknown / DNA-binding protein | 5647550..5647597 | 5647610..5647746 | 5647571..5647746 |
| 222 | ← ⇐ ← | transcription elongation factor / membrane protein | 5400560..5400596 | 5400527..5400680 | 5400524..5400705 |
| 234 | ← ⇐ → | lacI-family transcriptional regulatory protein / sugar binding secreted protein | 6033473..6033508 | 6033535..6033602 | 6033463..6033766 |
| 261, tRNA ala | ← ⇐ ← |  | 3481788..3481828 | 3481839..3481913 | 3481618..3481912 |
| 270 | ← ⇒ ← | probable deoR-family transcriptional regulator / probable transmembrane efflux protein | 3506310..3506345 | 3506180..3506297 | 3506179..3506390 |
| 274 | ← ⇒ ← | xis, excisionase / possible ATP-binding protein | 5040755..5040792 | 5040566..5040754 | 5040566..5040809 |
| 329, 4.5S | ← ⇒ → | tRNA Ser / dnaZ, DNA polymerase III subunit gamma | 4457108..4457124 | 4456953..457133 | 4456952..4457144 |
| 341 | → ⇐← | membrane protein / unknown, AbaA regulator | 4375713..4375750 | 4375701..4375846 | 4375671..4375845 |
| 390 | ← ⇐ → | araC, septum site associated protein / morphological differentiation-associated protein | 3933460..3933499 | 3933503..3933702 | 3933089..3933701 |
| 389 | → ⇐ → | morphological differentiation-associated protein / oxidoreductase | 3934642..3934660 | 3934627..3934844 | 3934558..3934843 |
| 413.1 | ← ⇒ → | 16S rRNA / 413.2 | 3690965..3690993 | 3690627..3691375 | 3690537..3691374 |
| 413.2 | ← ⇒ → | 413.1 / trpS, araC regulator | 3691287..3691307 | 3690627..3691375 | 3690537..3691374 |
| 445 | → ⇒ → | yjr2, possible lipoprotein / rplJ, 50S ribosomal protein L10 | 5076348..5076372 | 5076150..5076441 | 5076121..5076440 |
| 458 | ← ⇐ → | lysR, WhiB / luxR | 5179501..5179518 | 5179346..5179728 | 5179345..5179727 |
| 462 | ← ⇐ → | whiB / hypothetical protein | 3321214..3321271 | 3321017..3321638 | 3321016..3321701 |
| 470, tmRNA | → ⇐ ← | possible transport protein / smpB, small protein B homologue | 3226486..3226537 | 3226539..3227049 | 3226434..3227050 |
| 472 | ← ⇐ ← | malate oxidoreductase / helicase | 3208570..3208598 | 3208642..3208817 | 3208422..3208887 |
| 482, tRNA lys | → ⇐ ← |  | 3079072..3079118 | 3079043..3079190 | 3078952..3079189 |
| 493 | ← ⇒ ← | citA / possible deoxyribonuclease | 2984164..2984186 | 2984116..2984277 | 2984070..2984296 |
| 528 | ← ⇒ ← | tRNA Met / tRNA Asn | 2647104..2647119 | 2646934..2647070 | 2646933..2647204 |
| 624 | ← ⇒ ← | purB / mug | 1765177..1765234 | 1765037..1765136 | 1765022..1765257 |
| 640, tRNA gly | ← ⇐ → |  | 4469848..4469872 | 4469880..4469955 | 4469647..4469954 |
| 676 | ← ⇒ → | glycine dehydrogenase / hypothetical | 1457996..1458009 | 1457688..1457993 | 1457665..1458078 |

* The double arrow represent the sRNA gene. The simple arrows indicate the flanking genes. The left-pointing arrows indicate the complementary strand, the right-pointing arrows the other strand.

** Rho-independent terminator

*** {Rutherford, 2000 #133}

Table SII. Further characteristics of the *Streptomyces* sRNAs predicted using the Rho-dependent termination.

| ID # | Strand* | Flanking genes annotations*** | C-rich stretch coordinates** | Conserved sequence coordinates | IGR coordinates |
| --- | --- | --- | --- | --- | --- |
| 60 | ← ⇒ → | secreted cellulase / questionable ORF, secreted cellulase | 7243996..7244023 | 7243744..7243995 | 7243727..7244040 |
| 544, M1 RNA | ← ⇐ ← | xlnB / integral membrane protein, araC | 2462879..2462900 | 2462901..2463220 | 2462879..2463222 |

* The double arrow represent the sRNA gene. The simple arrows indicate the flanking genes. The left-pointing arrows indicate the complementary strand, the right-pointing arrows the other strand.

** The C-rich stretch indicating the Rho-dependent transcription termination

*** {Rutherford, 2000 #133}

Table SIII. Experimental detection of expression of the predicted *S. coelicolor* sRNAs

| ID # | Internal primer for microarrays | Primer for rt-pcr 3’ reverse | Primer for PCR 5’ sense * |
| --- | --- | --- | --- |
| 4 | GTTGGTGGTCCAAGGAAAGACGCCCCACTTC | GTTCTCGTCGAGCGCTGG | CGTTGGTGGTCCAAGGAA |
| 17 | GGCGATCTGTAGCTGGTCCAGCTTAATCCAAAATG |  |  |
| 36 | GGCGATCTGTAGCTGGTCCAGCTTAATCCAAAATG |  |  |
| 60 | GAGAAAGTCGGTAACTCCTCTGTTCTTTGCCGTCAC | ATGAGCGACTCCTTGCAG | TGCTGGTTCCTCCTTGCG or T_(18)_VN |
| 73 | GAGTCTCCTCAAGGGGTAGCCGGTGGGG | GTCAAACCCCCCACGCCC | T_(18)_VN |
| 84 | GGCTTCGGACTTCTGGTCAGCAGTCAGC |  |  |
| 95 | TTCTGGTGATTGCGTGAAGTACGCC | GGCGTACTTCACGCAATCACCAGAAACGGAACTAA | GAGAAAGGGACGTGCGGC or T_(18)_VN |
| 96 | CTTGATCCCCAGAAACGTCGACGATGGAC |  |  |
| 114, 5S RNA | GGTTACATTTCGAACCCGGAAGCTAAGCCTTACAGC |  |  |
| 115 | GTGAGTACAAAGGAAGCGTCCGTTCCTTGAGAACTC | CTTCAAAGGAACCTCAAC | GATTGGGAATCGCCGAGA or T_(18)_VN |
| 116 | GAGCCATGGGTTACACGCTACTCGTTGCTCAG |  |  |
| 126 | AGGTCATTCTGGGTAGGGAGATCGGTCACCACTC |  |  |
| 146 | TCTCTCCTTGTGTGCAGGTGTTGCGTCCCACCGTAT | GCATTCCGAGACCCCGAA | T_(18)_VN |
| 155 | GACCGCTCCTGTCCGCGT | TGTGTCGTGATGTCGACA | GACCGCTCCTGTCCGCGT |
| 156 | GCATATACTGTGCCTACTGAACTTCTTCGCGACCTC | CCGGCGCAGAAAAAATCC | AATTCCACTCCCGGCCGC or T_(18)_VN |
| 200 | CTTGGTACCGGGTTCTTTTCTTGTCCCGAGGCTCCT | GCCGCGGAACCCCACCCG | GGGGTGGTCTTCCTCGTC or T_(18)_VN |
| 222 | CTTATGTCCTCCCCCTTTGGCCACTGAATTGTTAG | CTAACAATTCAGTGGCCAAAGGGGGAGGACATAAG | TACCTGCCACCTGACCTG or T_(18)_VN |
| 234 | AGCCGCATGTCGACACAGCCCGT |  |  |
| 261, tRNA ala | ACCTCGTTCGCATCGAGGGGGTCTGGGGTTCAAATC |  |  |
| 270 | GAGGCTACGCCTGGTTGTCCGGAATGTAAATAGGA | TCCTATTTACATTCCGGA | GACCGTTCACCTCCGACC or T_(18)_VN |
| 274 | CTGCGTCATGTGCGTCATCTGCGTCATG | GCATGACGCAGATGACGC | T_(18)_VN |
| 329, 4.5S RNA | GAACATTACATGACTCCGAGGAGTGCTTGCGAACTC | CGCACCCGCCAGAGCCGA | GCGGAGAACATTACATGA or T_(18)_VN |
| 341 | TTGCTAACCGCCGATCTCTTGTTACCGTTCGAGTAG | CTACTCGAACGGTAACAAGAGATCGGCGGTTAGCAA | ACCGGTCACAACTCCCGG or T_(18)_VN |
| 390 | CTGAGAGGGCTCTCGGGTACACCATGGAAGTCAC | GAAGTGCGGAAAACCCAC | ATAGGCGCAGCCCATTCG or T_(18)_VN |
| 389 | AGTCCTTTGTACTACCGGTCCCGAGTAAGCGGAAGT | ACTTCCGCTTACTCGGGACCGGTAGTACAAAGGACT | ATCAGGCCGGGAACTTCG or T_(18)_VN |
| 413.1 | CTCAAGGAACGAACGCTTCCTTTGTACTCACCCTCT | GAGTCCGCTAGAGTCTCA | AATGTTTACCCGTAATCG or T_(18)_VN |
| 413.2 | GACTCTCGTCGAAGAGTGGTGAGTACTTGCGGAATC |  |  |
| 445 | GTTCACGTACGCTTCCACAGAAGCCAAAGACC |  |  |
| 458 | GGATACGGTGTCCACGACCGTACAACTACGATCTAA |  |  |
| 462 | GTTCACACAGGCACTGTCAACTCGATGACCGTTATG |  |  |
| 470, tm RNA | CTGATCTCAGGTAGCGAAGCGAGCCTCCTACTAAGG | TGGTGGAGATGGCGGGAATC | GGGGATGATCGGTTTCGACAG |
| 472 | GTCCAAGGTAACAAACGATCCCCGCAAGACAATTCC | GGAATTGTCTTGCGGGGATCGTTTGTTACCTTGGAC | GCGCCGGGCCGGGGGAGC or T_(18)_VN |
| 482, tRNA lys | GCTCAGTTGGTTAGAGCAGCTGACTCTTAATCAGCG |  |  |
| 493 | ACTTGGCTCAGGAGAGTGCACTCGGGGT |  |  |
| 528 | ATAGCCGCTCATACGATCTCCTGCCTCGGATGGTC | CGGGAACCGCGCGGCACG | ATAGCGGCGTGTCGCGTA or T_(18)_VN |
| 544, M1 | GTAAGGGTGAAACGGTGGTGTAAGAGACCACCAGT | GCAGACGAGTCGGGCTGT | AACGGCCACCCGGGGTGA or T_(18)_VN |
| 624 | AGGAGTGTCTCCTGTGAGGGCTGTGCCGGT | CACCCCGCCGTTCCCCCG | AGGAGTGTCTCCTGTGAG or T_(18)_VN |
| 640, tRNA gly | GTAGCTCAGTTGGTAGAGCGCAACCTTGCCAAGGT |  |  |
| 676 | CTCCCCCTCTGTCATCTCAACCTGAGAGCTTCAC | TCGGCCACTCGAAGTCCG | GACCTTCGAGGGGCACCA |

* T_(18)_VN primer was used after polyA-polymerization of 3’ end of cDNA product (N=A, C, G, or T; V=A, C, or G)

+ Predicted identity

Table SIV. Structure conservation of the predicted *Streptomyces* sRNAs using RNAz

| sRNA ID # | SVM RNA-class probability$ | Mean z-score* | Structure conservation index& |
| --- | --- | --- | --- |
| 4 | 1 | -4.19 | 0.91 |
| 17 | 1 | -3.15 | 0.81 |
| 36 | 0.98 | -2.08 | 0.72 |
| 73 | 0.91 | -1.73 | 0.67 |
| 84 | 0.59 | -1.00 | 0.58 |
| 95 | 0.7 | -1.43 | 0.94 |
| 96 | - |  |  |
| 114, 5S RNA | 1 | -4.32 | 0.64 |
| 115 | 0.99 | -2.34 | 0.99 |
| 116 | 1 | -3.17 | 0.84 |
| 126 | 1 | -5.19 | 0.98 |
| 146 | ? |  |  |
| 155 | 0.96 | -2.31 | 0.67 |
| 156 | 0.94 | -6.50 | 0.83 |
| 200 | 1 | -3.55 | 0.98 |
| 222 | 0.9 | -2.03 | 0.81 |
| 234 | ? |  |  |
| 261, tRNA ala | 0.99 | -2.63 | 1.00 |
| 270 | 0.58 | -0.87 | 0.95 |
| 274 | 1 | -3.54 | 0.73 |
| 329, 4.5S | 0.73, 0.84 | -1.67, -2.57 | 0.92, 0.52 |
| 341 | 1 | -3.83 | 0.83 |
| 390 | 1 | -4.19 | 0.98 |
| 389 | 1 | -3.10 | 0.91 |
| 413.1 | 1 | -3.17 | 0.98 |
| 413.2 | 1 | -3.20 | 0.77 |
| 445 | 0.97 | -2.00 | 0.92 |
| 458 | 0.67 | -1.79 | 0.66 |
| 462 | 0.99 | -2.28 | 0.62 |
| 470, tmRNA | 1 | -3.55 | 0.88 |
| 472 | 1 | -2.56 | 0.95 |
| 482, tRNA lys | 1 | -4.57 | 0.94 |
| 493 | 0.97 | -2.45 | 0.72 |
| 528 | 0.62 | -1.51 | 0.80 |
| 624 | 0.95 | -7.01 | 0.84 |
| 640, tRNA gly | 1 | -3.31 | 0.99 |
| 676 | 1 | -2.67 | 0.94 |
| 60 | 0.99 | -2.64 | 0.95 |
| 544, M1 RNA | 1 | -3.15 | 1.06 |

* z-score calculated by RNAz. A z-score is calculated as z = (m-μ)/σ, where μ and σ are the mean and standard deviations, resp., of the MFEs of comparable random samples. Negative z-scores indicate that a sequence is more stable than expected by chance.

& The SCI compares the consensus MFE E_A_ derived by RNAalifold to the average MFE of the individual sequences <E> (SCI = E_A_/<E>). The SCI will be high if the sequences fold together equally well as if folded individually.

$ In alignments with P>0.5 a functional RNA is predicted. The higher this value, the more confident is the prediction.
